# Cocaine induces sex-associated changes in lipid profiles of brain extracellular vesicles

**DOI:** 10.1007/s11064-021-03395-x

**Published:** 2021-07-10

**Authors:** Qwynn Landfield, Mitsuo Saito, Audrey Hashim, Stefanie Canals-Baker, Henry Sershen, Efrat Levy, Mariko Saito

**Affiliations:** a Division of Neurochemistry, Nathan S. Kline Institute for Psychiatric Research, Orangeburg, NY, USA; b Department of Psychiatry, New York University School of Medicine, New York, NY, USA; c Center for Dementia Research, Nathan S. Kline Institute for Psychiatric Research, Orangeburg, NY, USA; d NYU Neuroscience Institute, New York University School of Medicine, New York, NY, USA; e Department of Biochemistry & Molecular Pharmacology, New York University School of Medicine, New York, NY, USA

**Keywords:** cocaine, brain extracellular vesicles, lipid, GD1a ganglioside, sex difference

## Abstract

Cocaine is a highly addictive stimulant with diverse effects on physiology. Recent studies indicate the involvement of extracellular vesicles (EVs) secreted by neural cells in the cocaine addiction process. It is hypothesized that cocaine affects secretion levels of EVs and their cargos, resulting in modulation of synaptic transmission and plasticity related to addiction physiology and pathology. Lipids present in EVs are important for EV formation and for intercellular lipid exchange that may trigger physiological and pathological responses, including neuroplasticity, neurotoxicity, and neuroinflammation. Specific lipids are highly enriched in EVs compared to parent cells, and recent studies suggest the involvement of various lipids in drug-induced synaptic plasticity during the development and maintenance of addiction processes. Therefore, we examined interstitial small EVs isolated from the brain of mice treated with either saline or cocaine, focusing on the effects of cocaine on the lipid composition of EVs. We demonstrate that 12 days of noncontingent repeated cocaine (10 mg/kg) injections to mice, which induce locomotor sensitization, cause lipid composition changes in brain EVs of male mice as compared with saline-injected controls. The most prominent change is the elevation of GD1a ganglioside in brain EVs of males. However, cocaine does not affect the EV lipid profiles of the brain in female mice. Understanding the relationship between lipid composition in EVs and vulnerability to cocaine addiction may provide insight into novel targets for therapies for addiction.

## Introduction

Cocaine, a highly addictive and powerful stimulant, triggers various cellular and molecular alterations in the rewarding systems, and cocaine addiction is viewed as a disorder of neuroplasticity (1–4). While even a single injection of cocaine potentiates synaptic strength of excitatory inputs on the ventral tegmental area (VTA) dopaminergic neurons by altering AMPA receptor composition (5), repeated cocaine exposure causes more stable changes in the brain at the molecular and cellular levels that potentially underlie addictive behavior. For instance, cocaine increases cAMP response element-binding protein and ΔfosB levels in a class of medium spiny neurons in nucleus accumbens (NAc) of mice, which is related to increased locomotor and rewarding responses to cocaine (6). Cocaine can also induce structural plasticity by altering the complexity of dendritic branching as well as the number and size of dendritic spines on neurons in several brain regions (4). Associated with the addiction process, cocaine induces various physiological and pathological effects, such as increased immune reactivity, neuroinflammation (7, 8), and neurodegeneration (9, 10).

It was shown that extracellular vesicles (EVs), including exosomes and microvesicles (also termed ectosomes), are involved in many physiological and pathological processes in the brain. Functions of EVs include the removal of unnecessary cellular components and the transfer of biomolecules (proteins, lipids, and nucleic acids) to other neighboring or distal cells to mediate intercellular communication critical in physiological processes, such as nerve regeneration and synaptic function (11–16). However, some biomolecules carried by EVs can propagate or regulate neuroinflammation and neurodegenerative diseases (12, 13, 15, 17–21). Recent studies also indicate that EVs play important roles in addiction processes (22). EVs and their cargoes may contribute to drug seeking, withdrawal and relapse behaviors induced by a variety of substances of abuse including cocaine (22, 23). It was also reported that miRNAs, which are transported by exosomes, are involved in cocaine addiction (24, 25). In vitro, cocaine exposure of glioblastoma cells increases EV release in addition to tunneling nanotubule formation (26), while cocaine reduces exosome levels secreted by microglial cells (BV2) (27). *In vivo*, acute cocaine injection induces EV secretion through interaction with sigma-1 receptors in the midbrain, and the secreted EVs contain 2-arachidonoylglycerol (2-AG), which is an endocannabinoid neuromodulator highly implicated in addiction processes (28). Also, cocaine self-administration was shown to reduce the internalization of neuronal exosomes, particularly into astrocytes in NAc, which may contribute to altered glutamatergic synaptic plasticity through dysregulation of glutamate transporter in astrocytes (29). Thus, EVs may play important roles in addiction processes, which needs to be explored.

In the present study, we examined EVs in the mouse brain after repeated cocaine injections. Specifically, we focused on lipid profiles of EVs. Involvement of various lipids such as sex steroids, prostaglandins, endocannabinoids (2-AG, anandamide), and sphingolipids in drug-induced synaptic plasticity during the development and maintenance of addiction processes has begun to be appreciated (9, 30). Functions of these lipids in the addiction process may be mediated by lipids in EVs as suggested in the case of 2-AG (28). Various physiological and pathological states, such as neuroinflammation and neurodegeneration, which are relevant to the effects of cocaine (7–10, 31) can also be regulated by EV lipids (32–34). In addition, several lipids, such as ceramide, sphingosine 1-phosphate, lysobisphosphatidic acid (LBPA), are involved in EV formation and secretion (35–39). However, studies to examine compositions and functions of lipids in EVs are very limited (40, 41). Furthermore, lipid analyses of EVs have been largely done in cultured cells, and lipid changes induced by cocaine in brain EVs have not been reported except for the effect on endocannabinoids (28).

Our study shows that repeated cocaine treatment induces changes in composition of lipids, particularly sphingolipids, in brain EVs of male mice, but not of female mice.

## Materials and Methods

### Animals

C57BL/6 mice used in the studies were purchased from the Jackson Laboratory (Bay Harbor, ME, USA), and bred and maintained at the Nathan S. Kline Institute animal facility on ad lib food and water at all times. All procedures were approved by the Nathan S. Kline Institute IACUC and were in accordance with NIH guidelines for the care and use of laboratory animals for the proper treatment of animals. Cocaine (cocaine-HCl, Sigma-Aldrich, St. Louis, MO, USA) (10 mg/kg in saline) or saline as control was injected intraperitoneally once a day for 12 days into 3-month-old male and female C57BL/6 mice. Mice were sacrificed by cervical dislocation 30 min after the final cocaine/saline injection, and right and left hemibrains without cerebella and olfactory bulbs were dissected and immediately frozen on dry ice and stored at −80° C until EV isolation.

### EV isolation

Small EV fractions were isolated from right hemibrains according to the method described in (42) except that the final density gradient was performed using OptiPrep gradient (Sigma-Aldrich) as described in (43). After the density gradient centrifugation, eight fractions were collected, rinsed in phosphate buffered saline (PBS), centrifuged at 100,000 × *g*, and the final pellet of each fraction was suspended in 30 μl of PBS. The protein amount of each fraction was determined using the Pierce BCA protein assay kit (Thermo Fisher Scientific, Waltham, MA, USA).

### Lipid analyses

Twenty μl of each EV fraction out of 30 μl of EV suspension obtained by the OptiPrep density gradient was lyophilized, and the dried powder was sonicated in 200 μl of the mixture of methyl-tert-butyl ether (MTBE) and methanol (1:1, V/V). The resultant fine powder suspension was centrifuged at 10,000 × *g* for 5 min, and the precipitate was re-extracted with 200 μl of the above solvent mixture. The supernatants were combined to obtain the total lipid extracts. The extracts were partitioned according to the method of Matyash, et al. (44). The lower aqueous-methanol phase containing gangliosides was evaporated to dryness, applied to high-performance thin layer chromatography (HPTLC) plates, and developed first with methanol/MTBE (1:1) until the solvent front reached 1 cm from the origin, then developed with chloroform/methanol/0.25% KCl (5:4:1) (45) till 9 cm from the origin (1cm below the top of the plate). The plates including five different concentrations of GM1 standards were stained with an orcinol reagent, scanned with the Gel Logic molecular imaging system (Carestream Health, Rochester, NY, USA) and analyzed by Multi Gauge ver.2.0 (Fujifilm USA Medical Systems, Stamford, CT, USA). The concentration of each ganglioside (GM1, GD1a, and GT1b) was calculated using GM1 standards. The upper organic MTBE phase obtained by the partition described above was evaporated to dryness and separated into neutral and acidic lipids using DEAE-Sephadex columns as described (46). The neutral lipid fraction including cholesterol ester (ChE), triacylglycerol (TAG), diacylglycerol (DAG), cholesterol, ceramide, hexosylceramide (HexCer), phosphatidylethanolamine (PE), phosphatidylcholine (PC), and sphingomyelin (SM) and five different concentrations of each lipid standard were applied on HPTLC plates and first developed with methanol/MTBE (1:1) until the solvent front reached 0.5 cm from the origin, then developed with chloroform/methanol/water (65:25:4) until the solvent front ascended to 4.5 cm from origin, and next with acetone/benzene/acetic acid/water (10:15:2.5:0.5) until 6.5 cm, and finally with hexane/MTBE/acetic acid (98:2:1) until 9 cm above origin. The acidic lipid fraction including free fatty acid (FA), n-acylphosphatidylethanolamine (NAPE), phosphatidic acid (PA), lysobisphosphatidic acid (LBPA), cardiolipin (CL), phosphatidylserine/phosphatidylinositol (PS/PI) and five different concentrations of each lipid were developed on HPTLC first with chloroform until 5 cm from origin, then methanol/MTBE (1:1) till 5.5 cm, next with acetone/benzene/acetic acid/water (20:30:4:1) till 8 cm, and finally with hexane/MTBE/acetic acid (98:2:1) till 9 cm from the origin. (Since PS and PI were not separated well in this system, both bands were measured together.) After development, plates were first dipped in 20 % methanol and then stained in 0.0001% primuline as described (47). Then, fluorescent lipid bands were scanned and analyzed as described above for ganglioside analyses. For total brain (without cerebella and olfactory bulbs) lipid analyses, lipids were extracted from left hemibrains by 20 ml per 1g wet weight of hemibrains of the mixture of MTBE and methanol (1:1, V/V) and were analyzed as described for EV lipids. Solvents (HPLC grade) were purchased from Thermo Fisher Scientific, and all other chemicals and HPTLC plates (#1056410001) were purchased from Sigma.

### Statistics

The comparisons of levels of each lipid between saline and cocaine groups (or between male and female groups) or the comparisons between fractions separated by OptiPrep density gradient were done by two-way mixed ANOVA using SPSS statistics software (version 24). For post hoc analysis, adjustment by Bonferroni’s multiple comparison test was used. For all analyses, *P* < 0.05 was considered statistically significant. Values are expressed as mean ± S.E.M. obtained from 5 male and 5 female animals in each group (cocaine and saline).

## Results

### Cocaine alters lipid composition of EVs in brains of male mice.

**Fig. 1.**
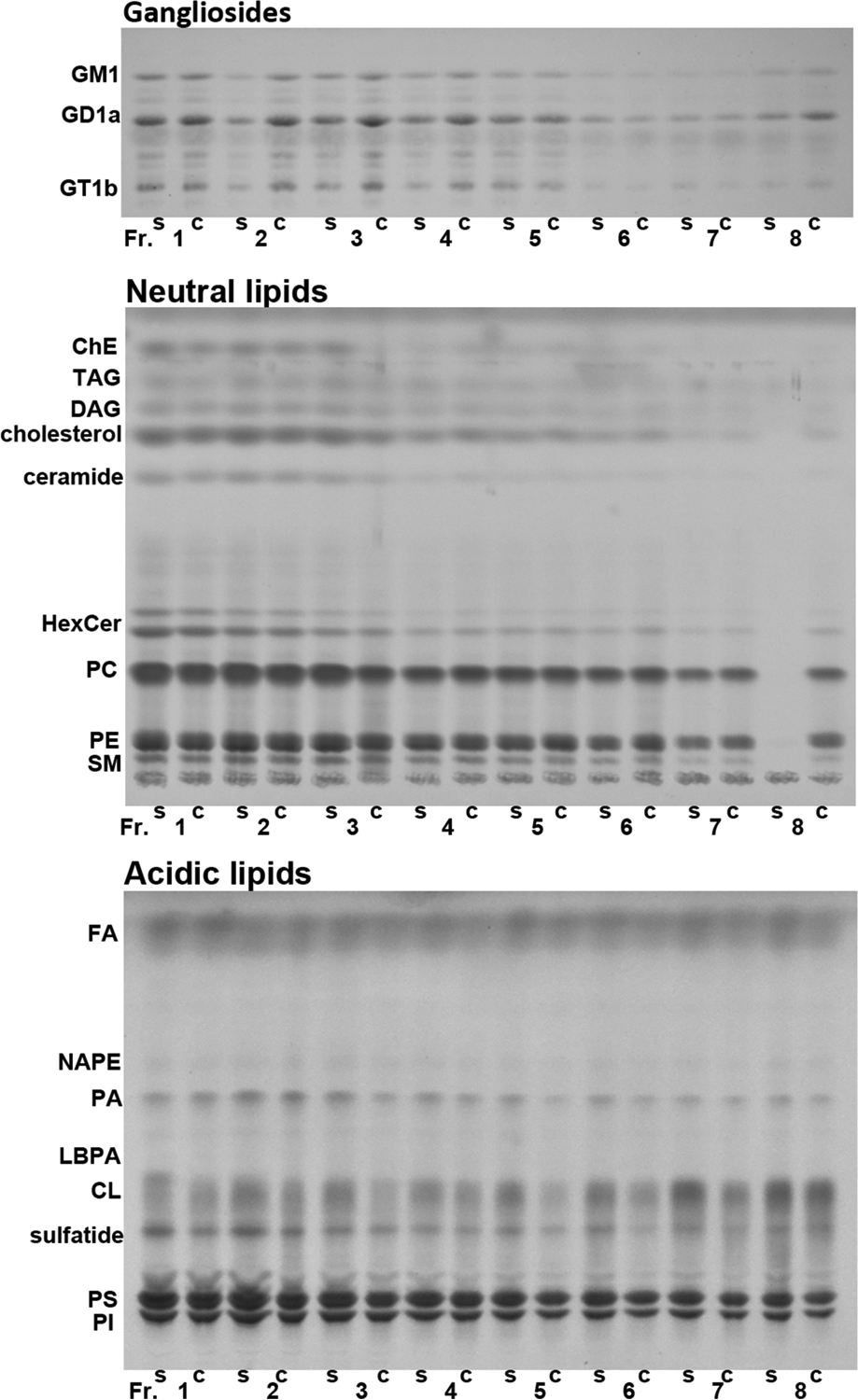
Representative images of lipids in EV fractions 1–8 isolated from male brains, separated on HPTLC. Total lipids extracted from each EV fraction were separated into three groups of lipids (gangliosides, neutral lipids, and acidic lipids), and each group of lipids was loaded on HPTLC plates. s: saline, c: cocaine.

**Fig. 2.**
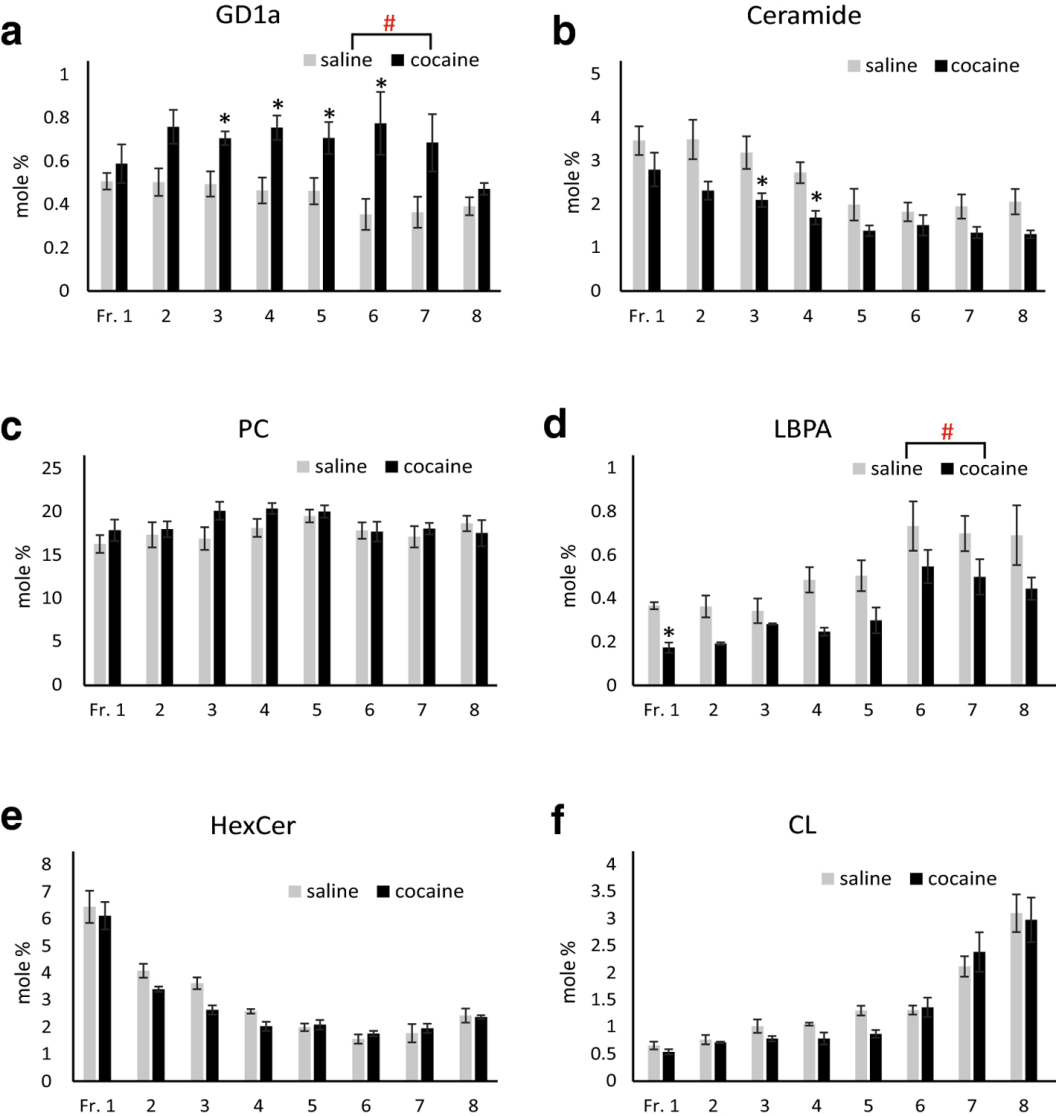
Effects of cocaine on GD1a, ceramide, PC, LBPA, HexCer, and CL of EV fractions isolated from male brains. Developed HPTLC plates were quantified using lipid standard developed simultaneously. For the analyses of gangliosides and acidic lipids, entire materials obtained from 20 μl of EVs were loaded on HPTLC, while one half of materials was loaded in the case of neutral lipids. This figure shows graphic presentations of results of GD1a, ceramide, PC, LBPA, HexCer, and CL listed in Table 1. Each lipid is presented as mean ± S.E.M. mole percent (mol%; n=5). ^#^Significant main effects of treatment (saline/cocaine) (p<0.05) were detected for GD1a and LBPA by 2-way mixed ANOVA. *indicates a significant difference (p<0.05) between saline and cocaine groups by pairwise comparisons with adjustment by Bonferroni multiple comparisons.

**Fig. 3.**
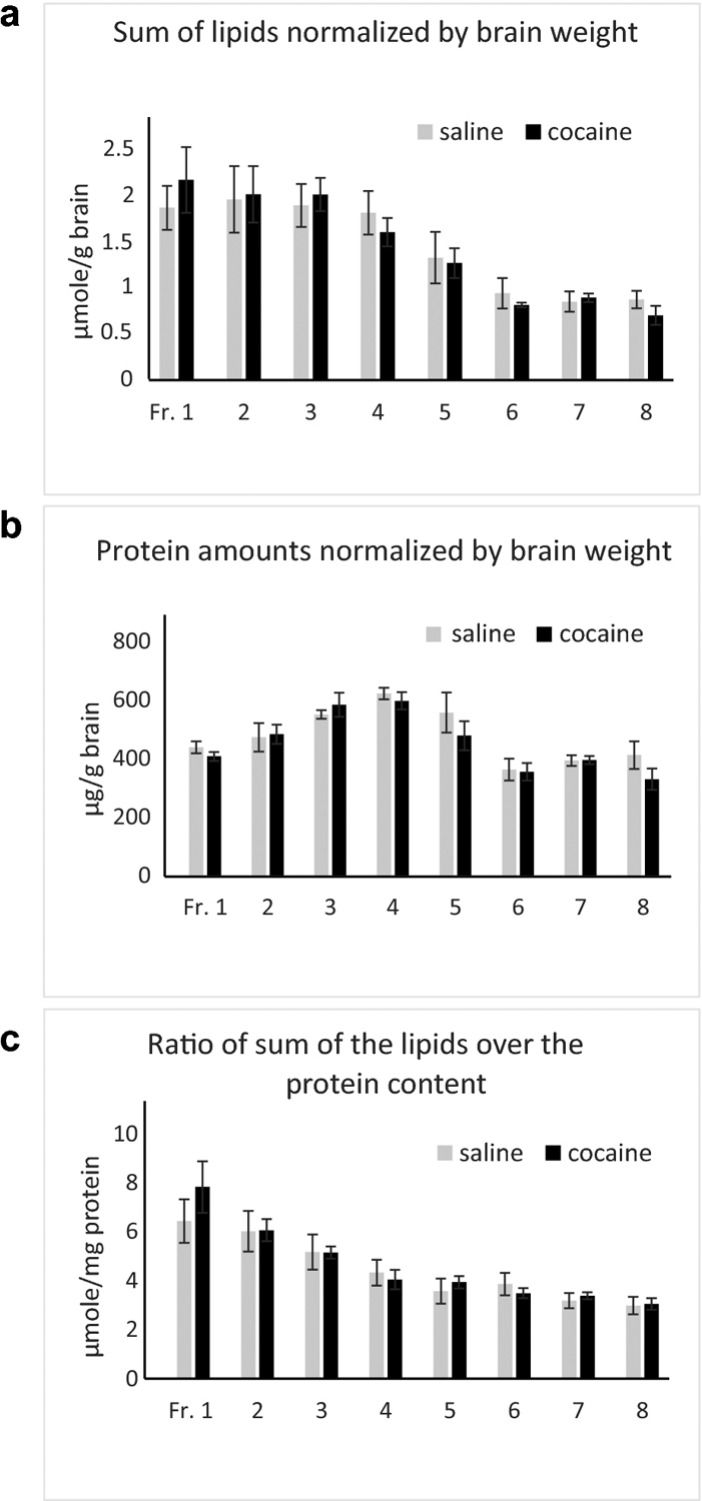
Effects of cocaine on amounts of total lipids and total proteins in EV fractions isolated from male brains. (a) Total lipid amounts (sum of all lipid amounts measured) are normalized by wet weight of hemibrain used and presented as mean ± S.E.M. (n=5, μmole/g brain wet weight). (b) Total protein amounts are normalized by wet weight of hemibrain used and presented as mean ± S.E.M. (n=5, μg/g brain wet weight). (c) Total lipid amounts (sum of all lipid amounts measured) normalized by EV protein amounts and presented as mean ± S.E.M. (n=5, μmole/mg protein).

Lipids are major components of EVs, and some lipids are involved in EV formation/secretion and cocaine addiction (28, 35–39). Therefore, we examined if repeated cocaine treatment, which induces locomotor sensitization (48), alters lipid profiles of EVs in male mouse brains. Small EVs were isolated from the right hemibrains and separated on an OptiPrep density gradient into 8 fractions (Fr.1–8), with Fr.1 having the lowest and Fr.8 the highest density. Our previous studies indicate that fractions 1–3, 4–6, and fraction 8 are enriched in small microvesicles, exosomes, and mitovesicles, respectively, judged by their sizes, densities, lipid and protein content, and electron microscopic analyses (43). Total lipid extracts from each EV fraction were separated into 3 groups (gangliosides, neutral lipids, and acidic lipids). Then, the whole ganglioside fraction (including GM1, GD1a, GT1b), one half of the neutral lipid fraction (including ChE, TAG, DAG, cholesterol, ceramide, HexCer, PC, PE, and SM), and the whole acidic lipid fraction (including FA, NAPE, PA, LBPA, CL, sulfatide, and PS/PI) were loaded in each lane of HPTLC plates and separated as described in Materials and Methods. Fig. 1 shows representative HPTLC plates developed for gangliosides, neutral lipids, and acidic lipids analyses. Table 1 shows compositions of major lipids of each brain EV fraction from male mice treated with saline or cocaine, expressed as mole percent (%) of the sum of all lipids analyzed. While we did not measure very minor lipid components, the lipids measured here represent more than 98% of the total brain lipids (49). Differences in each lipid content (expressed as mole percent) between treatments (cocaine and saline) and between EV fractions were tested using two-way mixed ANOVA (treatment as between and fraction as within factors). While significant interactions between treatment and fraction were detected only in NAPE, significant main effects of treatment (saline/cocaine) were found in GD1a [F(1,8)=8.76, p=0.018] and LBPA [F(1,8)=7.66, p=0.024]. Pairwise comparisons with adjustment by Bonferroni multiple comparisons indicated that cocaine significantly increased GD1a in Fr. 3 to 6 (p=0.022, 0.014, 0.050, and 0.048, respectively) and decreased LBPA in Fr. 1 (p=0.001). The data indicate that cocaine increases the proportion of GD1a ganglioside and decreases the proportion of LBPA. However, the level of LBPA in EVs was very low, just above the level of detection, consistent with what was reported for EVs isolated from cell lines [reviewed in Skotland et al. (50)] and from brain tissue (43). It is also noticed that ceramide levels were lower in the cocaine group (Table 1). While it did not reach the significant level by mixed ANOVA [F(1,8)=4.19, p=0.075], pairwise comparisons with adjustment by Bonferroni multiple comparisons indicated cocaine significantly reduced ceramide in Fr. 3 (p=0.044) and Fr. 4 (p=0.012). Significant main effects of fraction were detected in most of the lipids, indicating that lipid compositions are different among EV fractions, as clearly shown for HexCer [F(7,56)=60.97, p<0.001] and CL [F(7,56)=33.27, p<0.001] consistent with what we reported previously (43). Fig. 2 shows graphic presentations of results of the lipids that are showing (Table 1) distinct characteristics in response to cocaine or in the distributions among different EV fractions. Cocaine increased the level of GD1a (Fig. 2a) and decreased the level of ceramide (Fig. 2b) and LBPA (Fig. 2d). HexCer was enriched in lower density EV fractions (Fig. 2e), while CL was enriched in higher density EV fractions (Fig. 2f) without the effect of cocaine. The proportion of PC to other lipids was similar among EV fractions (Fig. 2c). The effect of cocaine was found for specific lipids following data calculation as mole percent (Table 1 and Fig. 2). However, sum of all lipids analyzed in each EV fraction (normalized to the wet weight of hemibrain and presented as μmole lipid per g brain) was not significantly different between the saline and cocaine treated males (Fig. 3a). In addition, the protein amounts of each EV fraction normalized to the wet weight of hemibrain showed no significant differences between the groups (Fig. 3b). Fig. 3c shows sum of all lipids analyzed in each fraction presented as μmole lipid per mg protein, which also was not significantly different between saline and cocaine groups. As expected, lipid/protein ratios were higher in lower density EV fractions. Thus, while cocaine does not affect the total amount of lipids in EVs, it changes the lipid compositions of EVs in male mouse brains.

### Cocaine does not alter the lipid compositions of EVs in brains of female mice.

**Fig. 4.**
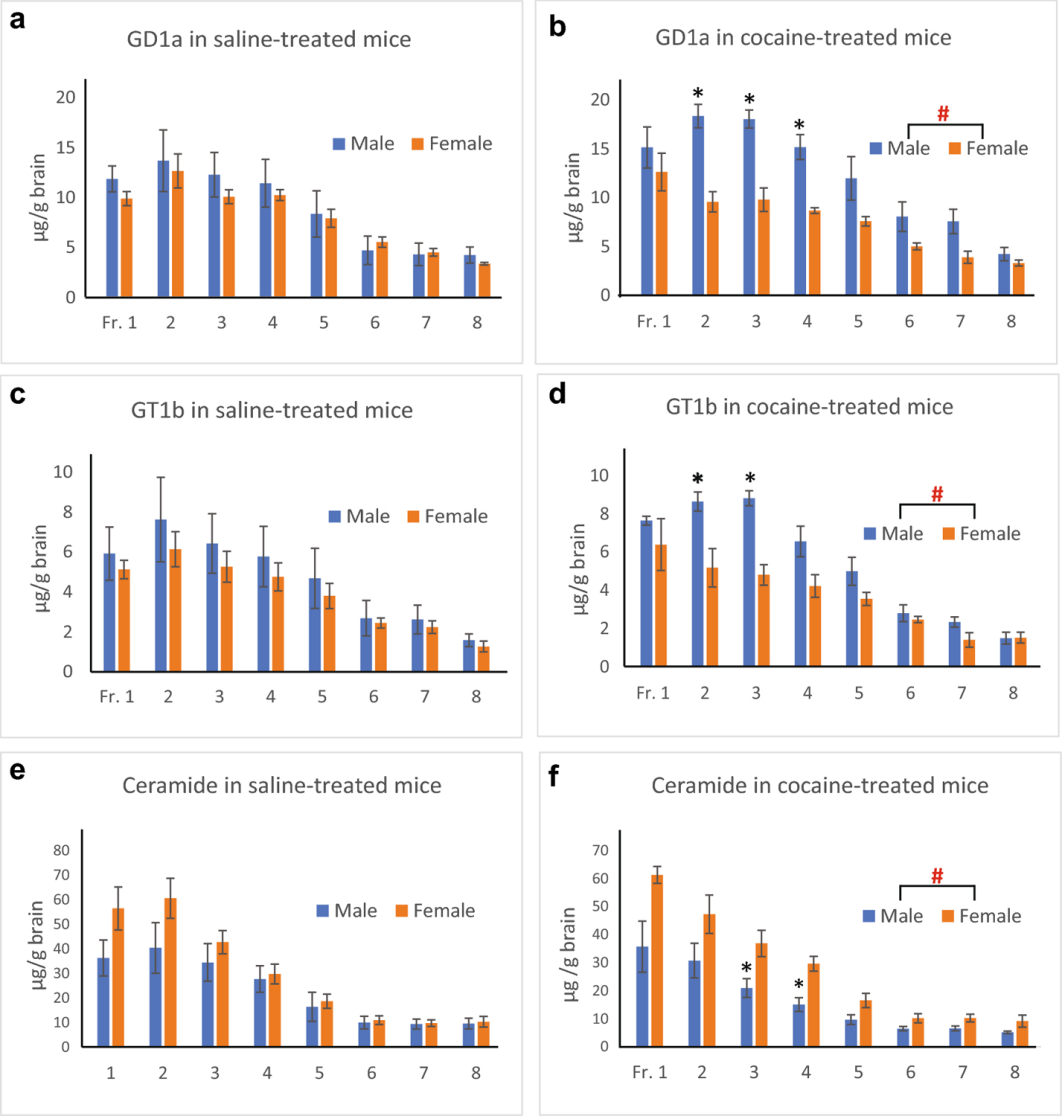
Comparisons of EV lipids between males and females with either cocaine or saline treatment. The content of GD1a (a, b), GT1b (c, d), and ceramide (e, f) of each EV fractions was compared between male and female mice treated with either saline (a, c, e) or cocaine (b, d, f). The lipid amounts are normalized by wet weight of hemibrain used and presented as mean ± S.E.M. (n=5, μg/g brain wet weight). ^#^Significant main effects of sex (p<0.05) were detected in GD1a, GT1b, and ceramide in the cocaine group, but not in the saline group by mixed ANOVA. *indicates a significant difference (p<0.05) between saline and cocaine groups by pairwise comparisons with adjustment by Bonferroni multiple comparisons.

Lipid analyses of EV fractions were also carried out on EVs isolated from brains of female mice injected with either saline or cocaine. Table 2 shows lipid compositions of each EV fraction presented as mole percent of the sum of all lipids analyzed. In contrast to male samples, no significant differences between the saline and cocaine groups were detected. By mixed ANOVA, no significant interaction between treatment and fraction and no significant main effects of treatment were found, while significant differences among EV fractions were observed in the same manner as for male samples. Also, the sum of all lipids analyzed in each EV fraction (normalized to the wet weight of hemibrain) was not significantly different between the saline and cocaine groups as indicated in the male samples. Fig. 4 shows the effects of cocaine treatment on GD1a and GT1b ganglioside and ceramide content (normalized by hemibrain wet weight) in EV fractions isolated from brains of male and female mice. Two-way mixed ANOVA indicates that while there are no significant differences in GD1a amounts between males and females in the saline group [F(1,8)=0.182, p=0.681] (Fig. 4a), there are significant differences between males and females in the cocaine group [F(1,8)=12.15, p=0.008], and pairwise comparisons with Bonferroni adjustment show that Frs.2, 3, and 4 are significantly different (Fig. 4b). Similarly, GT1b amounts are not different between males and females in the saline group [F(1,8)=0.317, p=0.589] (Fig. 4c), but are significantly different in the cocaine group [F(1,8)=8.831, p=0.018], and pairwise comparisons with Bonferroni adjustment show that Frs. 2 and 3 are significantly different (Fig. 4d). Also, ceramide levels are not significantly different between males and females in the saline group [F(1,8)=1.097, p=0.325] (Fig. 4e), but significantly different in the cocaine group [F(1,8)=8.331, p=0.02] and pairwise comparisons with Bonferroni adjustment show that Frs. 3 and 4 are significantly different (Fig. 4f). Thus, cocaine affects the lipid composition of EVs in a sex-dependent manner.

### Sphingolipids are highly concentrated in brain EVs.

The effect of cocaine on the lipid profiles of the hemibrains was also studied. In Table 3, the column with a header “brain” shows amounts of lipids of male left hemibrains presented as μg per mg protein. These values were not significantly different between the saline and cocaine groups by Student’s t test. The columns with headers Fr.1–8 in Table 3 show the ratios of each lipid amount (μg/mg protein) of EVs isolated from right hemibrains of male mice over each lipid amount (μg/mg protein) of left hemibrains of male mice to examine the enrichment of certain lipids in the EV fractions. Results indicate that gangliosides, ceramide, PS/PI, and fatty acids were highly enriched in lower to medium density-EV fractions both in saline and cocaine groups, although two-way mixed ANOVA indicates significantly higher fold changes in GD1a in EVs of the cocaine group compared to the control group (p<0.05) with a significant pairwise difference between saline and cocaine groups in Fr. 3 (p<0.05).

## Discussion

Previous studies indicate that EVs play important roles in addiction induced by a variety of substances of abuse including cocaine (22, 23). Lipids carried by EVs may participate in this addiction process (28), because involvement of various lipids, such as endocannabinoids and sphingolipids, in addiction has been recognized (9, 30, 51). In addition, lipids in EVs may affect various physiological and pathological states induced by cocaine, such as neuroinflammation and neurodegeneration (7–10, 31).

We analyzed the content of major lipids in EVs isolated from right hemibrains of male mice after 12 days of saline/cocaine (10 mg/kg) injections. Small EVs isolated from brains were separated into eight EV fractions (Fr.1-Fr.8) using OptiPrep density gradient, and lipids in each fraction were extracted and analyzed. Compared to lipid profiles of the left hemibrain, EV fractions, especially Frs.1–6, were enriched in gangliosides, ceramides, PS/PI, and FA in both the saline and cocaine groups (Table 3). Enrichment of these lipids in EVs agrees with previous studies of lipid analyses of exosomes secreted from various types of cultured cells (39, 52, 53). Enrichment of gangliosides and ceramides in exosomes was also found in our previous study in the brain of apolipoprotein E3 and apolipoprotein E4 targeted-replacement mice (54). In addition, it was suggested that there are EV subtypes defined by their lipid compositions (55). Mature oligodendrocytes are reported to release EVs containing galactosylceramide (GalCer) and sulfatide, which are abundant in myelin/oligodendrocytes (56), while gangliosides are enriched in exosomes secreted by primary cultured neurons, but not in exosomes secreted by primary cultured glia (57). Here we show that HexCers are specifically abundant in lower density fractions enriched in microvesicles (Table 1, Fig. 2e). The EVs containing HexCer (mainly GalCer) are likely derived from myelin/oligodendrocytes, while EVs enriched in gangliosides may derive from neurons. Therefore, lipid compositions of EVs are cell type-specific although there can be some lipids which are generally enriched in exosomes or microvesicles. Our results showed that various EVs with different lipid compositions are present in the brain, but EV fractions containing microvesicles or exosomes are enriched in sphingolipids, especially gangliosides and ceramide. Among the phospholipids, enrichment of PS/PI was the highest (Table 3). Similar enrichment in gangliosides, ceramide, and PS/PI in EV fractions was also observed in female brains (data not shown).

Our results showed that cocaine changed the lipid profiles of EV fractions isolated from male brains. Significant changes detected by mixed ANOVA were elevation in GD1a gangliosides and reduction in LBPA in EV fractions (Table 1, Fig. 2). However, cocaine did not induce any significant changes in lipid profiles of the total brain of the male mice (Table 3). It was reported that cocaine induces changes in lipidome of NAc: increases in glycosphingolipids, PC, PE, and a decrease in ceramide (58). We cannot rule out the possibility that the cocaine-induced changes in lipids are brain region-specific and cannot be detected when lipids in the whole brain are analyzed.

Cocaine-induced elevation in EV gangliosides may be related to the addiction process. GM1 was shown to enhance rewarding properties of cocaine, probably through elevation of brain derived neurotrophic factor (BDNF) (59, 60). Alternatively, EV gangliosides may exert neuroprotective reactions to alleviate cocaine’s adverse effects. Neuroprotective functions of gangliosides are well established (61, 62). While gangliosides in exosomes can enhance aggregation of amyloid β (Aβ) (34, 63) and α-synuclein (64), ganglioside-rich neuron-derived EVs are able to enhance Aβ clearance in a model of Alzheimer’s disease (57), and GM1 reduces α-synuclein toxicity in a model of Parkinson’s disease (PD) (65, 66). It was also shown that GM1 enhances autophagy-dependent removal of α-synuclein in a PD model (67). Since it was reported that α-synuclein is needed for the cocaine addiction process and cocaine-induced exosome release (68), the interaction between EV gangliosides and α-synuclein may be important in both cocaine-induced addiction and neurotoxicity. In addition to the ganglioside elevation, our studies indicated that cocaine reduced ceramide and LBPA in EVs isolated from male mouse brains. It was recognized that several lipids, such as ceramide, sphingosine 1-phosphate, and LBPA, are involved in EV formation and secretion (35–39). It appears that formation of intraluminal vesicles, released as exosomes when late endosomes/multivesicular bodies fuse with the plasma membrane, may be dependent on the ESCRT (the endosomal sorting complexes required for transport) pathway which needs LBPA or can be dependent on the ceramide pathway (69). The reduction in LBPA and ceramide in EVs from cocaine-treated male mice may affect intraluminal vesicles formation and exosome secretion, because we have observed a reduction in EV numbers measured by nano tracking analysis and in exosomes positive for the marker proteins, Alix and Tsg101, in cocaine-treated male mice (Barreto et al., manuscript in preparation). While cocaine exposure increases EV release by glioblastoma cells (26) and in the midbrain (28), cocaine reduces exosome levels secreted by microglial cells (BV2) (27). Since ganglioside GD1a, which is enriched in neurons, increased in EVs from cocaine-treated male mice in our experiments, cocaine may preferentially reduce the number of glia-derived EVs. Ceramide is also known as an apoptosis inducer, and ceramide-enriched exosomes can mediate cytokine-induced cell death in oligodendroglioma (41), and exosomes enriched in ceramide and prostate apoptosis response 4 (PAR-4) induce apoptosis in primary cultured astrocytes (32). Also, ceramide-enriched exosomes appear to exacerbate Alzheimer’s disease-related brain pathology *in vivo* (63). Therefore, EVs in the brain of male mice treated with cocaine, which have higher amounts of gangliosides and lower amounts of ceramides, may be more neuroprotective and less neurotoxic.

In contrast to male mice, cocaine did not induce any significant changes in lipid profiles of EVs isolated from female mouse brains (Table 2, Fig. 4). Multiple studies describe sex differences in the etiology of addiction of drugs including cocaine, and suggest that females are more sensitive to the motivated and rewarding properties of cocaine (70, 71). Clinical studies indicate that women develop substance use disorders more rapidly after initial use, and report shorter periods of abstinence during recovery, take higher doses, and experience more cravings (72–76). Similar sex differences in response to cocaine are seen in experimental animals (71, 75, 77, 78). In both humans and animals, the ovarian hormone estrogen seems to be a key biological factor contributing to the female vulnerability to cocaine (70, 71, 78–80). It has been indicated that estradiol increases VTA dopamine neuron activity and induces conformational changes in dopamine transporter, which augments cocaine-induced elevation in dopamine levels (81). Also, estradiol appears to mediate activation of metabotropic glutamate receptor type 5 through estrogen receptor activation, inducing synaptic plasticity in NAc, which is related to drug addiction and to underlining psychostimulant behavioral sensitization (82,83). Consistently, our previous studies (48) demonstrated that repeated non-contingent cocaine injections induced higher locomotor sensitization in females than in males.

Presently, it is unknown how cocaine induces lipid profile changes in EVs in the brain of males but not of females. One important possibility is that the ovarian hormone estrogen contributes to this sex difference. As described above, estrogen is a key factor involved in female vulnerability to cocaine (70, 71, 78–80). Cocaine-induced alterations in brain EV lipids may also be influenced by this hormone. Experiments using ovariectomized females with and without estradiol replacement will be our next step of this study.

Differences in cocaine-induced EV ganglioside content between males and females may cause differences in addiction processes. Our studies (Barreto et al., manuscript in preparation) show that cocaine increases α-synuclein in brain EVs from female mice, but not from male mice. α-Synuclein is a major component of Lewy bodies in PD (84), and GM1 ganglioside binds to α-synuclein and inhibits its accumulation and pathological aggregation, leading to attenuation of PD pathophysiology (65–67). Since the involvement of α-synuclein in the cocaine addiction process is implicated (68), male brain EVs with cocaine-induced increased levels of gangliosides may reduce levels and functions of α-synuclein, resulting in lower addiction responses observed in male mice.

In a human study, it was reported that the levels of major gangliosides, especially neuronal-enriched GD1a and GT1b gangliosides, were reduced in the substantia nigra of male PD subjects compared to the male controls, but there were no differences in these ganglioside levels between female PD subjects and the controls (85). Decreased ganglioside levels may be associated with higher α-synuclein accumulation, which would affect PD pathophysiology, although the lack of change in ganglioside levels in female PD subjects is currently difficult to explain. These studies and our present study suggest the importance of examining sex differences in ganglioside metabolism under various pathological conditions.

It is also possible that decreases in ceramide and LBPA in male brain EVs are related to decreased generation of EVs that carry addiction-stimulated molecules such as α-synuclein. Also, ceramide can cause glial activation and neuroinflammation (86, 87), which appear to enhance cocaine addiction (88, 89). Reduction in EV ceramide may provide male mice more resistance to cocaine addiction.

Thus, our studies indicate that repeated cocaine treatment changes lipid compositions of EVs isolated from male mouse brains. The increase in GD1a ganglioside and the decrease in ceramide may be neuroprotective reactions against adverse effects of cocaine. Additionally, decreases in ceramide and LBPA may suppress EV formation. These changes were not observed in female mice that are more vulnerable to the addictive process. The connection between cocaine-induced alterations in EV lipids and cocaine-induced neuroadaptation remains to be explored.

## Figures and Tables

**Table 1.** Cocaine affects the lipid compositions (mole percent) of EV fractions isolated from male brains

Lipid	Treatment				EV Fraction				
		1	2	3	4	5	6	7	8
**GM1**	Saline	0.20 ± 0.02	0.21 ± 0.02	0.18 ± 0.01	0.19 ± 0.02	0.17 ± 0.02	0.14 ± 0.01	0.15 ± 0.02	0.14 ± 0.01
	Cocaine	0.25 ± 0.04	0.32 ± 0.06	0.25 ± 0.03	0.30 ± 0.05	0.23 ± 0.05	0.17 ± 0.03	0.13 ± 0.02	0.17 ± 0.03
**GD1a**	Saline	0.51 ± 0.04	0.50 ± 0.06	**0.49 ± 0.06**	**0.47 ± 0.06**	**0.46 ± 0.6**	**0.35 ± 0.07**	0.36 ± 0.07	0.39 ± 0.05
	Cocaine	0.59 ± 0.09	0.76 ± 0.09	**0.71 ± 0.04**	**0.76 ± 0.06**	**0.71 ± 0.07**	**0.77 ± 0.15**	0.69 ± 0.13	0.47 ± 0.03
**GT1b**	Saline	0.27 ± 0.04	0.31 ± 0.06	0.29 ± 0.05	0.26 ± 0.05	0.29 ± 0.05	0.23 ± 0.05	0.26 ± 0.05	0.16 ± 0.02
	Cocaine	0.35 ± 0.05	0.42 ± 0.06	0.39 ± 0.04	0.38 ± 0.06	0.35 ± 0.04	0.30 ± 0.04	0.24 ± 0.03	0.18 ± 0.03
**HexCer**	Saline	6.44 ± 0.60	4.08 ± 0.26	3.62 ± 0.22	2.58 ± 0.09	1.99 ± 0.14	1.56 ± 0.17	1.77 ± 0.34	2.43 ± 0.29
	Cocaine	6.12 ± 0.51	3.40 ± 0.11	2.64 ± 0.19	2.03 ± 0.17	2.09 ± 0.18	1.76 ± 0.10	1.96 ± 0.17	2.37 ± 0.08
**Sulfatide**	Saline	2.06 ± 0.15	1.32 ± 0.20	1.03 ± 0.21	0.80 ± 0.13	0.82 ± 0.16	0.70 ± 0.12	0.87 ± 0.19	1.26 ± 0.20
	Cocaine	1.96 ± 0.24	1.50 ± 0.23	0.89 ± 0.07	0.90 ± 0.09	0.73 ± 0.09	0.88 ± 0.16	0.97 ± 0.11	1.31 ± 0.21
**Ceramide**	Saline	3.47 ± 0.33	3.50 ± 0.46	**3.19 ± 0.38**	**2.73 ± 0.24**	1.99 ± 0.37	1.82 ± 0.22	1.95 ± 0.28	2.06 ± 0.33
	Cocaine	2.80 ± 0.39	2.31 ± 0.24	**2.10 ± 0.18**	**1.69 ± 0.16**	1.39 ± 0.12	1.51 ± 0.24	1.34 ± 0.13	1.31 ± 0.10
**SM**	Saline	4.73 ± 1.00	4.57 ± 1.47	5.26 ± 0.72	5.46 ± 0.82	3.21 ± 0.32	2.75 ± 0.39	2.17 ± 0.39	2.26 ± 0.33
	Cocaine	3.30 ± 0.27	3.32 ± 0.27	4.11 ± 0.10	4.28 ± 0.08	4.09 ± 0.17	3.22 ± 0.24	2.52 ± 0.32	2.36 ± 0.15
**PC**	Saline	16.25 ± 1.02	17.31 ± 1.46	16.88 ± 1.31	18.12 ± 1.3	19.49 ± 0.74	17.82 ± 0.95	17.08 ± 1.22	18.63 ± 0.89
	Cocaine	17.85 ± 1.22	17.96 ± 0.93	20.09 ± 1.04	20.35 ± 0.63	20.00 ± 0.71	17.68 ± 1.13	18.01 ± 0.66	17.52 ± 1.52
**PE**	Saline	10.36 ± 0.40	10.53 ± 0.59	10.50 ± 0.53	11.36 ± 0.49	10.21 ± 2.04	13.40 ± 0.71	14.13 ± 0.72	14.13 ± 0.37
	Cocaine	10.44 ± 0.59	10.68 ± 0.70	12.08 ± 0.60	12.75 ± 0.80	12.62 ± 0.59	12.88 ± 0.56	14.64 ± 0.55	12.93 ± 1.46
**PS/PI**	Saline	7.52 ± 0.34	8.53 ± 0.96	9.81 ± 0.24	9.35 ± 0.33	10.47 ± 0.44	9.72 ± 0.20	10.15 ± 0.48	9.55 ± 0.59
	Cocaine	7.18 ± 1.26	7.81 ± 0.41	7.41 ± 0.48	8.11 ± 0.95	7.96 ± 1.20	7.16 ± 0.75	8.28 ± 0.77	7.50 ± 1.04
**PA**	Saline	0.43 ± 0.07	0.53 ± 0.09	0.59 ± 0.04	0.74 ± 0.06	0.73 ± 0.07	1.06 ± 0.14	1.31 ± 0.36	0.69 ± 0.19
	Cocaine	0.60 ± 0.04	0.76 ± 0.03	0.60 ± 0.08	0.70 ± 0.08	0.81 ± 0.10	1.16 ± 0.28	0.67 ± 0.10	1.06 ± 0.23
**LBPA**	Saline	**0.37 ± 0.02**	0.36 ± 0.05	0.34 ± 0.06	0.49 ± 0.06	0.51 ± 0.07	0.73 ± 0.11	0.70 ± 0.08	0.69 ± 0.14
	Cocaine	**0.17 ± 0.02**	0.19 ± 0.01	0.28 ± 0.00	0.25 ± 0.02	0.30 ± 0.06	0.55 ± 0.08	0.50 ± 0.08	0.45 ± 0.06
**CL**	Saline	0.66 ± 0.07	0.76 ± 0.09	1.01 ± 0.12	1.05 ± 0.03	1.30 ± 0.09	1.31 ± 0.08	2.12 ± 0.19	3.10 ± 0.39
	Cocaine	0.54 ± 0.05	0.71 ± 0.02	0.78 ± 0.05	0.78 ± 0.11	0.87 ± 0.07	1.36 ± 0.18	2.38 ± 0.37	2.98 ± 0.46
**NAPE**	Saline	0.14 ± 0.04	0.09 ± 0.02	0.12 ± 0.05	0.18 ± 0.06	0.11 ± 0.02	0.31 ± 0.09	0.36 ± 0.11	0.21 ± 0.04
	Cocaine	0.07 ± 0.02	0.21 ± 0.06	0.07 ± 0.01	0.09 ± 0.01	0.15 ± 0.02	0.19 ± 0.02	0.23 ± 0.02	0.23 ± 0.02
**Cholesterol**	Saline	33.59 ± 0.74	32.96 ± 2.35	33.65 ± 0.92	32.09 ± 1.47	29.35 ± 1.95	24.20 ± 1.25	21.92 ± 1.62	22.16 ± 0.55
	Cocaine	34.83 ± 1.10	36.05 ± 1.39	33.53 ± 2.13	32.35 ± 0.69	29.59 ± 1.24	23.00 ± 3.05	23.70 ± 2.96	23.76 ± 3.31
**ChE**	Saline	0.73 ± 0.10	0.81 ± 0.13	1.19 ± 0.21	1.25 ± 0.19	0.84 ± 0.25	0.98 ± 0.16	1.02 ± 0.20	1.02 ± 0.20
	Cocaine	1.60 ± 0.44	1.25 ± 0.47	2.16 ± 0.59	1.90 ± 0.85	1.98 ± 0.97	3.75 ± 1.77	3.23 ± 1.39	2.24 ± 1.22
**FA**	Saline	8.18 ± 0.79	9.44 ± 2.45	7.70 ± 1.29	8.64 ± 1.30	11.82 ± 1.61	16.42 ± 2.22	18.12 ± 3.38	16.06 ± 1.83
	Cocaine	7.64 ± 0.99	8.14 ± 0.89	7.87 ± 1.50	8.11 ± 0.59	10.31 ± 0.50	16.17 ± 2.41	13.69 ± 1.62	16.78 ± 3.92
**TAG**	Saline	1.90 ± 0.59	1.58 ± 0.58	1.61 ± 0.58	1.76 ± 0.43	1.72 ± 0.54	2.40 ± 0.73	1.94 ± 0.69	1.76 ± 0.60
	Cocaine	1.23 ± 0.27	1.72 ± 0.31	1.54 ± 0.12	1.48 ± 0.25	2.05 ± 0.32	2.79 ± 0.62	2.13 ± 0.31	2.22 ± 0.41
**DAG**	Saline	2.23 ± 0.05	2.65 ± 0.35	2.58 ± 0.20	2.57 ± 0.16	4.56 ± 1.04	4.10 ± 0.30	3.75 ± 0.30	3.32 ± 0.20
	Cocaine	2.45 ± 0.13	2.53 ± 0.16	2.52 ± 0.17	2.80 ± 0.42	3.73 ± 0.39	4.73 ± 0.68	4.75 ± 0.63	4.23 ± 0.78

Mole percent of each lipid in EV fractions 1 to 8 from male brains are presented as mean ± S.E.M. (n=5). Significant main effects of treatment (saline/cocaine) (p<0.05) were detected by 2-way mixed ANOVA in GD1a and LBPA (highlighted in yellow). Pairwise comparisons with adjustment by Bonferroni multiple comparison test show that cocaine increased GD1a in Frs. 3 to 6, decreased LBPA in Fr. 1, and decreased ceramide in Frs. 3 and 4 significantly (highlighted in red).

**Table 2.** Cocaine does not affect the lipid compositions (mole perecent) of EV fractions isolated from female brains

Lipid	Treatment				EV Fraction				
		1	2	3	4	5	6	7	8
**GM1**	Saline	0.18 ± 0.01	0.20 ± 0.04	0.19 ± 0.02	0.18 ± 0.02	0.15 ± 0.02	0.13 ± 0.01	0.12 ± 0.00	0.10 ± 0.00
	Cocaine	0.23 ± 0.04	0.18 ± 0.02	0.18 ± 0.01	0.18 ± 0.02	0.16 ± 0.01	0.13 ± 0.01	0.10 ± 0.01	0.12 ± 0.01
**GD1a**	Saline	0.37 ± 0.02	0.46 ± 0.06	0.39 ± 0.02	0.42 ± 0.02	0.42 ± 0.06	0.38 ± 0.04	0.33 ± 0.03	0.27 ± 0.02
	Cocaine	0.47 ± 0.07	0.42 ± 0.06	0.43 ± 0.05	0.37 ± 0.02	0.42 ± 0.03	0.37 ± 0.05	0.26 ± 0.04	0.32 ± 0.06
**GT1b**	Saline	0.22 ± 0.03	0.26 ± 0.05	0.25 ± 0.04	0.22 ± 0.03	0.23 ± 0.04	0.19 ± 0.02	0.17 ± 0.03	0.12 ± 0.03
	Cocaine	0.27 ± 0.06	0.27 ± 0.07	0.25 ± 0.04	0.23 ± 0.04	0.22 ± 0.02	0.20 ± 0.02	0.10 ± 0.02	0.17 ± 0.05
**HexCer**	Saline	3.89 ± 0.32	2.62 ± 0.33	1.68 ± 0.41	1.86 ± 0.33	1.31 ± 0.08	1.35 ± 0.09	1.40 ± 0.10	1.43 ± 0.10
	Cocaine	3.64 ± 0.31	2.52 ± 0.32	2.14 ± 0.17	1.69 ± 0.11	1.62 ± 0.17	1.04 ± 0.11	1.21 ± 0.19	1.36 ± 0.06
**Sulfatide**	Saline	1.63 ± 0.23	1.08 ± 0.10	0.97 ± 0.16	1.01 ± 0.15	0.96 ± 0.14	0.89 ± 0.16	1.25 ± 0.22	1.42 ± 0.17
	Cocaine	1.50 ± 0.17	1.23 ± 0.07	1.03 ± 0.09	0.98 ± 0.15	0.87 ± 0.13	1.09 ± 0.20	1.13 ± 0.19	1.38 ± 0.22
**Ceramide**	Saline	4.92 ± 0.61	5.04 ± 0.47	3.87 ± 0.32	2.84 ± 0.35	2.19 ± 0.24	1.71 ± 0.25	1.54 ± 0.20	1.87 ± 0.31
	Cocaine	5.41 ± 0.40	4.84 ± 0.60	3.82 ± 0.38	3.22 ± 0.27	2.12 ± 0.30	1.68 ± 0.19	1.64 ± 0.23	1.91 ± 0.31
**SM**	Saline	2.56 ± 0.44	5.72 ± 3.13	7.04 ± 3.15	5.83 ± 2.96	6.27 ± 3.08	6.02 ± 3.36	5.91 ± 3.35	5.50 ± 3.37
	Cocaine	5.57 ± 0.42	5.84 ± 2.98	5.30 ± 2.66	4.57 ± 1.89	5.91 ± 2.85	6.17 ± 3.69	5.15 ± 3.10	5.11 ± 3.17
**PC**	Saline	15.79 ± 2.42	15.85 ± 2.31	14.61 ± 2.00	15.26 ± 1.95	15.62 ± 2.10	15.29 ± 1.49	15.74 ± 1.85	14.05 ± 1.42
	Cocaine	13.94 ± 2.71	15.16 ± 2.90	14.93 ± 3.09	13.79 ± 3.19	15.15 ± 2.08	14.29 ± 1.51	14.60 ± 1.55	13.72 ± 1.29
**PE**	Saline	9.62 ± 1.12	8.84 ± 1.10	8.42 ± 0.93	8.94 ± 1.32	9.61 ± 1.24	10.04 ± 1.71	10.11 ± 1.71	9.70 ± 1.45
	Cocaine	9.74 ± 1.67	10.93 ± 1.71	10.58 ± 1.66	9.99 ± 1.67	10.90 ± 1.89	10.41 ± 2.05	11.28 ± 1.91	10.61 ± 1.80
**PS/PI**	Saline	7.25 ± 1.21	7.57 ± 0.88	7.01 ± 0.77	7.16 ± 1.14	8.56 ± 1.48	8.74 ± 1.37	7.94 ± 1.69	6.87 ± 1.16
	Cocaine	6.73 ± 0.85	8.37 ± 0.89	8.85 ± 0.88	7.53 ± 1.09	8.39 ± 1.15	9.83 ± 1.39	8.19 ± 1.49	6.65 ± 1.01
**PA**	Saline	0.57 ± 0.11	0.63 ± 0.05	0.71 ± 0.13	0.90 ± 0.03	0.87 ± 0.05	0.72 ± 0.05	0.72 ± 0.04	0.76 ± 0.20
	Cocaine	0.63 ± 0.05	0.63 ± 0.09	0.70 ± 0.05	0.79 ± 0.10	0.78 ± 0.08	0.95 ± 0.16	0.86 ± 0.12	0.99 ± 0.20
**LBPA**	Saline	0.16 ± 0.04	0.18 ± 0.02	0.18 ± 0.03	0.21 ± 0.06	0.21 ± 0.01	0.24 ± 0.04	0.38 ± 0.11	0.22 ± 0.04
	Cocaine	0.15 ± 0.02	0.19 ± 0.02	0.26 ± 0.02	0.23 ± 0.07	0.32 ± 0.10	0.32 ± 0.09	0.18 ± 0.01	0.40 ± 0.12
**CL**	Saline	0.75 ± 0.06	0.93 ± 0.13	0.84 ± 0.11	1.14 ± 0.10	1.32 ± 0.15	1.88 ± 0.22	2.51 ± 0.34	2.97 ± 0.62
	Cocaine	0.81 ± 0.04	0.95 ± 0.04	1.07 ± 0.03	1.14 ± 0.13	1.30 ± 0.13	1.92 ± 0.17	2.62 ± 0.47	2.99 ± 0.47
**NAPE**	Saline	0.06 ± 0.01	0.09 ± 0.02	0.10 ± 0.01	0.11 ± 0.02	0.11 ± 0.01	0.14 ± 0.02	0.18 ± 0.03	0.18 ± 0.04
	Cocaine	0.08 ± 0.02	0.12 ± 0.02	0.09 ± 0.01	0.10 ± 0.02	0.14 ± 0.04	0.13 ± 0.02	0.14 ± 0.02	0.27 ± 0.06
**Cholesterol**	Saline	35.59 ± 2.20	36.67 ± 1.54	37.03 ± 1.73	36.92 ± 1.32	33.72 ± 1.47	30.33 ± 1.41	28.27 ± 1.54	28.51 ± 2.44
	Cocaine	37.95 ± 1.55	32.27 ± 4.12	33.63 ± 2.29	37.78 ± 2.16	33.47 ± 1.45	29.98 ± 1.58	29.48 ± 2.41	28.05 ± 1.86
**ChE**	Saline	1.50 ± 0.52	1.65 ± 0.56	3.09 ± 0.27	1.79 ± 0.43	2.21 ± 0.56	1.81 ± 0.72	2.34 ± 0.73	2.08 ± 0.74
	Cocaine	1.76 ± 0.65	1.74 ± 0.55	2.72 ± 0.26	1.54 ± 0.28	2.35 ± 0.55	1.65 ± 0.70	2.50 ± 0.64	2.31 ± 1.08
**FA**	Saline	7.86 ± 0.75	7.36 ± 0.83	8.49 ± 1.17	10.04 ± 1.22	10.04 ± 1.06	13.46 ± 1.32	14.63 ± 1.93	16.07 ± 1.77
	Cocaine	6.49 ± 0.52	9.73 ± 2.21	9.38 ± 0.83	9.75 ± 0.93	9.93 ± 0.94	13.33 ± 1.09	13.14 ± 1.72	16.08 ± 2.13
**TAG**	Saline	1.62 ± 0.34	1.57 ± 0.28	1.78 ± 0.17	1.90 ± 0.44	2.24 ± 0.69	2.49 ± 0.62	2.05 ± 0.35	2.64 ± 0.51
	Cocaine	1.90 ± 0.54	1.65 ± 0.26	1.78 ± 0.17	2.02 ± 0.50	2.18 ± 0.67	1.99 ± 0.34	2.30 ± 0.39	2.38 ± 0.42
**DAG**	Saline	3.31 ± 0.63	3.40 ± 0.68	2.91 ± 0.42	3.34 ± 0.62	4.02 ± 0.83	4.05 ± 0.68	3.79 ± 0.49	4.71 ± 0.83
	Cocaine	3.15 ± 0.30	2.91 ± 0.46	3.09 ± 0.27	4.13 ± 0.70	3.84 ± 0.65	4.27 ± 0.83	4.79 ± 0.93	4.64 ± 0.79

Mole percent of each lipid in EV fractions 1 to 8 from female brains are presented as mean ± S.E.M. (n=5).

**Table 3.** Enrichment of lipids in EV fractions compared to total brain

Lipid	Treatment	Brain				EV fraction				
			1	2	3	4	5	6	7	8
**GM1**	Saline	0.6 ± 0.0	37.7 ± 5.9	38.4 ± 7.5	26.7 ± 3.8	24.6 ± 4.6	17.5 ± 3.2	15.3 ± 0.9	13.4 ± 1.1	12.2 ± 0.7
	Cocaine	0.6 ± 0.1	48.3 ± 2.0	49.1 ± 7.0	37.7 ± 4.6	30.1 ± 4.2	23.9 ± 3.8	15.3 ± 2.3	11.7 ± 1.1	12.5 ± 3.1
**GD1a**	Saline	0.8 ± 0.1	53.3 ± 6.2	53.3 ± 11.2	**43.7 ± 8.5**	35.2 ± 7.2	29.1 ± 6.3	24.3 ± 6.0	20.8 ± 5.3	20.2 ± 3.3
	Cocaine	0.7 ± 0.1	81.3 ± 9.8	67.0 ± 15.8	**69.0 ± 4.1**	56.4 ± 5.0	52.8 ± 6.9	51.4 ± 11.5	43.6 ± 8.7	28.2 ± 2.9
**GT1b**	Saline	0.3 ± 0.0	62.6 ± 14.2	69.0 ± 18.8	53.6 ± 13.0	41.4 ± 10.8	37.6 ± 9.6	31.4 ± 8.1	29.7 ± 8.1	18.3 ± 3.5
	Cocaine	0.4 ± 0.0	78.6 ± 4.2	60.5 ± 15.1	64.6 ± 6.3	46.6 ± 6.2	42.0 ± 3.9	32.3 ± 3.8	24.3 ± 2.2	13.4 ± 4.0
**HexCer**	Saline	43.5 ± 5.4	8.5 ± 1.7	5.0 ± 0.9	3.6 ± 0.4	2.2 ± 0.2	1.4 ± 0.3	1.3 ± 0.3	1.2 ± 0.3	1.6 ± 0.3
	Cocaine	42.3 ± 4.8	10.2 ± 2.0	4.5 ± 0.4	2.6 ± 0.2	1.7 ± 0.2	1.7 ± 0.2	1.2 ± 0.1	1.4 ± 0.1	1.4 ± 0.1
**Sulfatide**	Saline	7.5 ± 1.0	15.0 ± 0.9	8.7 ± 0.6	5.6 ± 0.6	3.8 ± 0.2	3.1 ± 0.3	3.0 ± 0.3	3.0 ± 0.5	3.3 ± 0.9
	Cocaine	6.2 ± 0.2	20.6 ± 0.8	12.5 ± 1.2	7.1 ± 0.6	5.2 ± 0.8	4.1 ± 0.4	4.5 ± 0.9	4.7 ± 0.6	7.4 ± 1.7
**Ceramide**	Saline	1.7 ± 0.4	73.0 ± 15.3	70.8 ± 16.7	55.1 ± 12.9	38.5 ± 7.4	24.2 ± 7.0	22.9 ± 4.4	20.4 ± 4.3	20.5 ± 4.2
	Cocaine	1.1 ± 0.2	117.0 ± 27.1	85.6 ± 16.2	48.4 ± 6.0	35.0 ± 5.5	27.4 ± 3.7	26.6 ± 5.1	22.9 ± 2.9	22.6 ± 3.1
**SM**	Saline	35.0 ± 2.8	5.8 ± 0.8	5.4 ± 1.3	5.4 ± 0.5	4.6 ± 0.3	2.5 ± 0.5	2.4 ± 0.5	1.6 ± 0.4	1.6 ± 0.3
	Cocaine	34.0 ± 3.0	5.5 ± 0.7	4.7 ± 0.5	4.4 ± 0.2	3.7 ± 0.3	3.5 ± 0.3	2.4 ± 0.1	1.9 ± 0.3	1.7 ± 0.2
**PC**	Saline	121.7 ± 3.7	7.0 ± 1.3	7.0 ± 1.3	5.9 ± 1.1	5.2 ± 0.8	4.5 ± 0.7	4.5 ± 0.6	3.6 ± 0.5	3.9 ± 0.5
	Cocaine	124.4 ± 8.0	9.1 ± 1.6	7.7 ± 1.0	6.1 ± 0.7	5.2 ± 0.5	5.0 ± 0.5	3.9 ± 0.2	3.8 ± 0.2	3.2 ± 0.4
**PE**	Saline	121.4 ± 3.8	4.0 ± 0.4	3.8 ± 0.4	3.3 ± 0.4	3.0 ± 0.3	2.2 ± 0.6	3.2 ± 0.4	2.8 ± 0.3	2.9 ± 0.4
	Cocaine	118.4 ± 3.9	5.3 ± 0.9	4.5 ± 0.6	3.6 ± 0.4	3.3 ± 0.4	3.1 ± 0.2	2.8 ± 0.2	3.1 ± 0.2	2.3 ± 0.3
**PS/PI**	Saline	15.6 ± 0.9	24.7 ± 3.6	25.7 ± 4.1	25.5 ± 3.1	20.6 ± 2.7	18.9 ± 2.6	19.1 ± 2.2	16.5 ± 2.0	11.5 ± 2.9
	Cocaine	15.4 ± 0.3	25.7 ± 2.1	23.1 ± 1.1	19.4 ± 0.6	16.2 ± 1.6	15.5 ± 1.9	12.6 ± 1.2	14.3 ± 1.5	9.8 ± 1.9
**PA**	Saline	2.7 ± 0.4	7.8 ± 2.2	9.2 ± 2.4	7.9 ± 1.0	8.2 ± 0.9	6.7 ± 0.9	10.3 ± 1.3	9.9 ± 2.2	3.9 ± 1.3
	Cocaine	2.8 ± 0.4	11.7 ± 1.1	12.3 ± 0.9	7.4 ± 1.2	6.9 ± 0.5	8.0 ± 0.8	10.6 ± 3.0	5.8 ± 0.9	10.7 ± 2.7
**LBPA**	Saline	1.1 ± 0.1	16.6 ± 2.3	14.8 ± 3.3	12.0 ± 3.5	13.7 ± 2.7	11.4 ± 1.8	22.4 ± 3.9	13.9 ± 3.1	11.6 ± 3.1
	Cocaine	0.9 ± 0.0	11.2 ± 1.9	9.3 ± 1.0	10.4 ± 1.8	8.9 ± 1.2	11.5 ± 2.0	16.0 ± 2.8	12.8 ± 2.7	12.0 ± 2.4
**CL**	Saline	9.3 ± 0.3	6.9 ± 1.5	7.3 ± 1.1	7.7 ± 0.6	7.0 ± 0.6	7.0 ± 0.6	7.9 ± 1.0	10.9 ± 1.8	11.3 ± 3.2
	Cocaine	9.1 ± 0.2	6.4 ± 0.5	7.5 ± 0.7	6.2 ± 0.4	5.0 ± 0.1	5.4 ± 0.6	7.4 ± 1.0	13.0 ± 2.2	13.3 ± 1.4
**NAPE**	Saline	1.3 ± 0.1	6.0 ± 0.5	3.8 ± 0.3	4.0 ± 0.9	4.8 ± 1.0	3.9 ± 1.3	7.0 ± 1.4	7.3 ± 1.4	3.6 ± 0.1
	Cocaine	1.4 ± 0.1	5.5 ± 2.1	7.3 ± 1.7	2.8 ± 0.5	2.8 ± 0.4	4.8 ± 0.7	5.1 ± 0.7	5.4 ± 0.3	6.1 ± 0.7
**Cholesterol**	Saline	156.9 ± 5.9	5.6 ± 0.7	5.0 ± 0.7	4.3 ± 0.5	3.4 ± 0.5	2.6 ± 0.3	2.1 ± 0.3	1.8 ± 0.3	1.9 ± 0.3
	Cocaine	155.9 ± 6.8	6.4 ± 0.8	5.5 ± 0.5	4.1 ± 0.4	3.3 ± 0.3	2.9 ± 0.4	2.3 ± 0.3	2.0 ± 0.3	1.6 ± 0.3
**ChE**	Saline	5.7 ± 0.6	13.1 ± 3.3	11.7 ± 3.1	13.0 ± 1.8	9.6 ± 1.9	5.9 ± 2.1	10.4 ± 3.1	8.4 ± 2.0	7.1 ± 1.9
	Cocaine	6.9 ± 0.3	13.6 ± 4.5	11.2 ± 4.0	10.9 ± 3.2	8.2 ± 4.0	8.0 ± 3.9	12.5 ± 5.9	10.5 ± 4.5	11.1 ± 5.2
**FA**	Saline	3.9 ± 0.3	37.0 ± 4.4	36.5 ± 5.6	26.6 ± 2.6	25.0 ± 1.6	27.9 ± 0.8	43.1 ± 3.1	38.5 ± 4.2	26.6 ± 6.4
	Cocaine	4.5 ± 0.4	36.5 ± 4.7	32.8 ± 3.5	25.1 ± 5.1	20.7 ± 2.2	25.9 ± 2.0	36.8 ± 7.1	29.2 ± 3.4	40.5 ± 9.9
**TAG**	Saline	4.6 ± 0.2	18.4 ± 4.3	14.2 ± 3.7	12.7 ± 3.6	12.5 ± 1.9	10.6 ± 2.5	15.0 ± 3.4	9.9 ± 2.8	8.7 ± 2.5
	Cocaine	4.7 ± 0.2	15.8 ± 2.0	21.0 ± 3.5	14.1 ± 1.1	11.0 ± 2.5	14.7 ± 2.5	17.4 ± 3.7	13.1 ± 2.0	16.0 ± 4.0
**DAG**	Saline	11.1 ± 1.5	7.6 ± 1.0	7.9 ± 0.4	7.1 ± 0.9	6.0 ± 0.8	7.9 ± 1.3	8.5 ± 1.3	6.2 ± 0.4	6.0 ± 0.9
	Cocaine	11.0 ± 1.7	10.3 ± 1.3	8.1 ± 0.3	6.6 ± 0.6	6.4 ± 1.3	7.9 ± 0.9	9.0 ± 1.6	8.7 ± 1.3	8.3 ± 1.8

The column named “brain” shows amounts of lipids extracted from male left hemibrains presented as mean ± S.E.M. μg/mg protein (n=5). In the columns named “Fr.1–8”, the amount of each lipid (μg/mg protein) in each EV fraction isolated from the male right hemibrain was divided by the lipid amount (μg/mg protein) of the male left hemibrain. Values are expressed as fold changes (means ± S.E.M., n=5). Two-way mixed ANOVA indicates significant differences in GD1a between cocaine and the control groups (p<0.05, highlighted in yellow) with a significant pairwise difference in Fr. 3 (p<0.05, highlighted in red).

## Data Availability

All data and material are available in the article or upon request.
